# Food Insecurity: Child Care Programs’ Perspectives

**DOI:** 10.1007/s10995-021-03320-2

**Published:** 2022-01-08

**Authors:** Tracy E. Noerper, Morgan R. Elmore, Rachel B. Hickman, Madison T. Shea

**Affiliations:** 1 Department of Nutrition, Lipscomb University, One University Park Drive, Nashville, TN 37204 USA; 2 Texas Tech University, 2500 Broadway Avenue, Lubbock, TX 79409 USA; 3 East Tennessee State University, 1276 Gilbreath Drive, Johnson City, TN 37614 USA

**Keywords:** Child care, Food insecurity, Child and adult care food program, Children, Nutrition

## Abstract

**Background:**

Households experiencing "food insecurity" have limited access to food due to a lack of money or resources. Poor nutrition, from food insecurity, can impact physical and cognitive development of children. Study objectives were to document the prevalence of Tennessee child care programs screening for food insecurity, explore differences between programs receiving child and adult care food program (CACFP) funding and those screening for food insecurity, and understand possible burdens food insecurity places on child care families as perceived by child care program directors.

**Methods:**

In this cross-sectional study of licensed Tennessee child care programs, a 10-question survey and four-question follow-up survey were electronically distributed. Analysis included descriptive statistics, a chi-square of programs receiving CACFP funds and screening for food insecurity, and themes analysis of open-ended responses.

**Results:**

The average child care program enrollment (N = 272) was 80.16 with programs serving mostly preschoolers (98.53%) and toddlers (91.91%). Over half (56.99%) of programs reported they received CACFP funding, yet only 9.19% screen for food insecurity. Chi-square analysis found that programs receiving CACFP funds differ significantly on whether they screen households for food insecurity $\chi$ ^2^ (1, n = 237) = 16.93, *p* ≤ 0.001. Themes analysis (n = 41) revealed that many child care program directors do not view food insecurity as a burden for families.

**Conclusions:**

Child care programs receiving CACFP funds are more likely to screen families for food insecurity than programs who do not. Programs indicate a willingness to include food insecurity screening questions on child care paperwork.

## Significance

*What is already known on this subject?*

The term “food insecurity” means a household has limited access to adequate food for healthy living. During 2020, 13.8 million U.S. households experienced food insecurity. Children can be negatively impacted by poor nutrition due to food insecurity. Validated screening tools can help identify households at risk for food insecurity.

*What does this study add?*

A paucity of data exists on the role that child care programs play in reducing household food insecurity. This study examines child care program directors’ perspectives of food insecurity, the extent child care programs are screening for food insecurity, and any burdens that food insecurity may place on child care households.

## Introduction

In 2020, 89.5% of U.S. households reported having enough food to maintain a healthy quality of life, while the remaining 10.5% struggled with adequate food at some point during the year (Coleman-Jensen et al., 2021). Households lacking access to enough food for an active, healthy life are termed “food insecure” by the United States Department of Agriculture (USDA) (Coleman-Jensen et al., 2021). Food insecurity is a difficult reality for many children. Children and adults experienced food insecurity in 7.6% of U.S. households during 2020, meaning that households were unable at times to access and provide adequate, nutritious food for their children (Coleman-Jensen et al., 2021).

*Healthy* *People* 2020 guidelines are national public health objectives intended to improve the health of all Americans (Healthy People, 2020). One *Healthy* *People* 2020 objective, aimed solely at children, seeks to “eliminate very low food security among children.” Baseline data for this objective found that nationally, “1.3 percent of households with children had very low food security” with a goal of only “0.2 percent of households with children experiencing very low food security” (Healthy People, 2020). Very low food security indicates that normal eating patterns were disrupted and food intake reduced due to insufficient money or other resources (Coleman-Jensen et al., 2021).

To identify households at risk for food insecurity, health professionals support the practice of screening (Gattu et al., 2019; Hager et al., 2010). Food insecurity screening tools are available for use in a variety of medical and community settings (Makelarski et al., 2017; Nikolaus et al., 2019). For example, the validated screening tool known as the “Hunger Vital Sign™,” (Gattu et al., 2019) asks:

1. Within the past 12 months, I worried whether our food would run out before I got money to buy more*. *(*often true, sometimes true, never true*)
2. Within the past 12 months, the food I bought just didn’t last and I didn’t have money to get more. (*often true, sometimes true, never true*)

If a person indicates an “often true” or “sometimes true” response to either or both questions, then a referral to a food assistance program should be initiated. The “Hunger Vital Sign™” screening tool has not been validated specifically in child care settings.

In the early years of children’s growth and development, it is critical they receive proper nutrition. Children growing up in households with insufficient food can be at risk for behavioral, social, educational, psychological, and developmental complications (Alaimo et al., 2001; Shankar et al., 2017). Young children in food insecure households have higher odds of fair or poor health compared with children in food secure households (Cook et al., 2004; Drennen et al., 2019). Food insecure families may be forced to purchase lower-cost, less nutritious foods to stretch their food dollars. Less costly foods can be heavily processed, calorie-dense and lack macro- or micronutrients (Drewnowski, 2004). Evidence suggests that a reduced level of fruit and vegetable availability and frequency of consumption declines as the level of household food insecurity status worsens (Kendall et al., 1996). A reduction in the access and availability of healthy foods can have long-term consequences for children’s growth and development. For example iron, found in foods like meat, spinach, raisins, and iron-fortified cereal, is an important mineral needed for cognitive and behavioral growth (Baker & Greer, 2010). Food insecure children are “significantly more likely to have iron-deficiency anemia compared to food secure children” (Skalicky et al., 2006). This type of nutritional deficiency could result in a lasting negative impact on a child’s developmental and cognitive status (Lozoff et al., 1991).

National programs, like the supplemental nutrition assistance program (SNAP) or the national school lunch program (NSLP), are designed to improve the nutritional status of low-income individuals (Holben & Marshall, 2017). A similar program, the child and adult care food program (CACFP), provides reimbursement funds to centers who serve nutritious meals and snacks to adults and children (USDA, 2020b). The CACFP differs from SNAP or the NSLP in that it provides reimbursements directly to programs for the meals and snacks they serve. Specifically for child care settings, participation in the CACFP is “open to most child care providers” but reimbursement rates differ depending upon factors like the type of care (e.g. child care home versus child care center), licensure, for-profit or nonprofit status, income level of the neighborhood or income of the child’s household (Heflin et al., 2015). For example, home-based child care providers are eligible to receive tiered CACFP meal reimbursements for children in their program based on a combination of factors like the “provider’s neighborhood, the provider’s income and the income of the families that place children in the provider’s care” (Gordon et al., 2011). Child care centers would also be eligible to participate in the CACFP, but their reimbursement rates vary based on the enrolled child’s income eligibility for paid, free or reduced price meals (USDA, 2020d).

In 2019, 4795 national locations participated in the CACFP (USDA, 2020a). The Tennessee CACFP reported a daily attendance of 87,704 and served 39,245,951 total meals in 2019 (USDA, 2020c). Nationally in 2019, 96.0% of CACFP meals served were to child care centers and day care homes combined, with 80.9% of those meals being served to free or reduced price qualifying centers (USDA, 2020c). These national and state data underscore the important role of the CACFP in improving the “health and development of children, especially low-income children” (Gordon et al., 2011).

Participation in the CACFP program has been linked to improvements in household food insecurity. Child care programs participating in the CACFP have seen a “small reduction (4.19%) in the risk of household food insecurity” by providing children nutrient-dense meals and snacks (Heflin et al., 2015). Increasing the food supply to children “may not only affect the food security status of the participating child but also increase the food consumed by adults in the household” (Heflin et al., 2015). Additionally, Korenman and colleagues (2013) found that “CACFP participation moderately increases consumption of milk and vegetables, and may also reduce the prevalence of overweight and underweight among low-income children.” Besides those benefits, CACFP reimbursements could potentially lower child care costs for families since meals and snacks are offered to enrolled children and reimbursements are provided directly to child care programs (USDA, 2019).

## Objectives

Study objectives were to document the prevalence of Tennessee child care programs screening for food insecurity, explore differences between programs receiving child and adult care food program (CACFP) funding and those screening for food insecurity, and understand possible burdens food insecurity places on child care families as perceived by child care program directors.

## Methods

Approval of the study was obtained by the university’s Institutional Review Board and all researchers were trained in ethical research procedures prior to initiation of the study. A cross-sectional study of Tennessee child care programs was conducted. Programs were recruited from a Tennessee Department of Human Services state-wide listing of licensed child care programs and contacted by email with a consent opportunity for study participation. Inclusion criteria were if the child care program participant self-identified as either the program director or assistant director. All participants provided consent prior to participating in the study.

The researchers created an initial 10-question survey and four-question follow-up survey. Eight subject matter experts (SME) in nutrition, child care and social work conducted face and content validity assessments for both surveys. Content validity ratio (CVR) was calculated for both surveys, and minor survey edits were made based on SME feedback.

The initial electronic questionnaire included questions about the type of child care program, location (region), enrollment and ages of children, ethnic and racial categories of the children, whether the program received CACFP meal reimbursements, and if the program conducted food insecurity screenings of their child care households. The USDA definition of food insecurity was provided on the survey along with a screening question example for context (Coleman-Jensen et al., 2021). Ethnic and racial survey categories were taken directly from the Tennessee CACFP monthly reporting form and age group classifications were taken exactly from the *Tennessee Licensure Rules for Child Care Agencies* (Tennessee Department of Human Services, 2019).

Any participant indicating they would be willing to take part in a follow-up survey provided their email address. The four-question follow-up electronic survey included questions about the burdens food insecurity puts on households, resources needed for households experiencing food insecurity, and whether child care programs would consider adding food insecurity screening questions to household admissions paperwork. SurveyMonkey® (SurveyMonkey Inc., 1999–2020) was used to distribute both the initial and follow-up surveys with four weeks to complete each survey.

The researchers imported survey data into JMP® data analysis software (JMP, 2019) and computed means, frequencies, and standard deviations (N = 272). A chi-square analysis was performed to explore the association between child care programs receiving CACFP funds and programs who screen their child care households for food insecurity. Screening method examples provided by child care directors were extracted directly from survey data and reported. Data analysis of open-ended, follow-up survey responses was approached with a relativist ontological position meaning that reality is socially constructed, seen through many views, and is influenced by a variety of social factors (e.g., culture, language, etc.) (Creswell & Poth, 2016; Swift & Tischler, 2010). To increase trustworthiness of the open-ended survey response data analysis, researchers individually identified themes and coded text responses. The researchers then discussed as a group the coded text segments and themes (Attride-Stirling, 2001) for three topics: additional food insecurity information, burdens of food insecurity on child care families, and resources needed for food insecure child care families. Once the researchers reached consensus and achieved saturation of data analysis, themes analysis was concluded.

## Results

Validity results revealed that 100% of subject matter experts (N = 8) agreed with face validity for both the initial and follow-up survey. The content validity ratio (CVR) calculation for the initial 10-question survey was 0.80, meeting the 0.78 minimum CVR threshold (Pennington, 2003). The four-question follow-up survey CVR was 0.5, which did not meet the 0.78 minimum CVR threshold.

After validity analysis, the researchers distributed the electronic survey to 1,799 licensed Tennessee child care programs. Four hundred twenty participants accessed the survey, a 23.35% response rate, with 364 (86.67%) consenting and 56 (13.33%) declining. Participants meeting inclusion criteria and providing complete data sets were used for statistical analysis (N = 272).

Characteristics of the child care sample show that the average child care program enrollment was 80.16 children (*SD* = 152.30), a median of 52, and range from 4 to 1,500 (Table 1). The majority of child care program enrollees were non-Hispanic/Latino (*M* = 95.27%, *SD* = 11.81). White (*M* = 69.86%, *SD* = 36.26) and Black (*M* = 27.75, *SD* = 36.10) children were the largest racial groups enrolled in the programs. Child care centers made up 73.16% of the sample (n = 199), 15.44% were group child care homes (n = 42), and 7.72% were Family Child Care Homes (n = 21).

**Table 1:** Characteristics of Tennessee child care programs (N = 272)

Characteristic	*M*	*SD*
Average child care program enrollment	80.16	152.30
Ethnicity		
% Non-Hispanic or Latino	95.27	11.81
% Hispanic or Latino	4.73	11.81
Race		
% White	69.86	36.26
% Black or African American	27.75	36.10
% Asian	1.39	3.56
% Native Hawaiian or other Pacific Islander	0.66	6.32
% American Indian or Native American	0.34	1.59
	n	%
Type of child care program		
Child care center (13 or more children)	199	73.16
Group child care home (8 to 12 children)	42	15.44
Family child care home (7 or fewer children)	21	7.72
Other	9	3.31
Drop-in daycare	1	0.37
Child care program region		
Mid-Cumberland	70	25.74
Shelby	47	17.28
Southeast	29	10.66
South central	28	10.29
Upper Cumberland	28	10.29
East	27	9.93
Southwest	25	9.19
Upper East	18	6.62
Child care programs receiving CACFP funds		
Yes	155	56.99
No	116	42.64
Not sure	1	0.37
Child care programs screening for food insecurity		
Yes	25	9.19
No	213	78.31
Not sure	34	12.50

CACFP is the child and adult care food program. Food insecurity is a limited access to adequate food by a lack of money and other resources (Coleman-Jensen et al., 2021)

Child care programs served a variety of age groups including infants, toddlers, preschoolers and school-age children (Table 1). Age groups were analyzed as either “yes” or “no” since a program could serve more than one age group. Preschool children comprised the largest group served in 268 (98.53%) programs, followed by 250 (91.91%) serving toddlers, 185 (68.01%) serving infants, and 99 (36.40%) serving school-age children (Table 2).

**Table 2:** Age groups of children served in child care programs (N = 272)

	Yes N (%)	No N (%)
Infant (6 weeks to 12 months)	185 (68.01)	87 (31.99)
Toddler (13 months to 30 months)	250 (91.91)	22 (8.09)
Pre-school (at least 31 months and not entered kindergarten)	268 (98.53)	4 (1.47)
School-age (kindergarten to 17 years of age)	99 (36.40)	173 (63.60)

Counts are for rows

More than half (56.99%) of child care programs reported they received CACFP funds versus 42.64% of programs who did not (Table 1). When asked if child care programs conducted any type of food insecurity screening, 78.31% (n = 213) indicated “no”, while 9.19% (n = 25) reported “yes” (Table 1). Thirty-four programs (12.5%) were unsure if they conducted screenings. Programs who screened for food insecurity shared screening examples which included verbal conversations with families about food needs, evaluation of written information from household applications (e.g., income data, SNAP participation, food-related questions on paperwork), and direct observations of children (Table 3). Over 2500 children in 13 child care programs were screened for food insecurity either verbally or through observations made by child care directors. Approximately 1100 children were screened via a written method. Written screenings were the most frequently reported method used with “toddlers” and “preschoolers” being the most screened child care type. There was no indication that geographic region markedly influenced a certain screening method. Each of the child care programs who screened (Table 3) indicated they received CACFP funds except two. The two programs not receiving CACFP funds were both located in the “East” state region, served a combined total of 40 “Infants”, “Toddlers” and “Pre-schoolers” and were a “Child Care Center” and a “Group Care Child Home.”

**Table 3:** Summary of child care programs who report screening for food insecurity (n = 25)

Method of food insecurity screening reported by child care directors	Child care program enrollment		Child care program type	Age groups of children served	Geographic region
	n	*M* (SD)			
Verbal screenings	2251	321.6 (525.6)			
“During face-to-face orientation, staff partner with families to discuss any insecurities…”			Other	PS	Upper Cumberland
“Speak about WIC and refer to DHS for SNAP.”			Family child care home	T, PS	South Central
“Verbal conversation questions.”			Group child care home	I, T, PS, SA	East
“We ask how often certain foods are consumed on a daily/weekly basis…. meals eaten per day.”			Other	PS	Upper East
“In the classroom during lunch time food conversations are discussed.”			Child care center	PS, SA	Shelby
“We let them know of healthy recipes…where to get free meals…and provide a meal if needed.”			Family child care home	I, T, PS	Shelby
“We tell parents about the church food pantry.”			Child care center	T, PS, SA	Southwest
Written screenings	1103	91.9 (104.5)			
“Q & A forms regarding the child’s eating habits.”			Child care center	I, T, PS	Shelby
“During our application process there are questions asked ….”			Child care center	I, T, PS	Upper East
“It is not an in-depth screening… on enrollment parents complete forms for participation in the food program…The standard forms included socio-economic level…as well as participation in SNAP programs. However, no in-depth screening is done.”			Child care center	I, T, PS, SA	Mid Cumberland
“Food application for Our Daily Bread of TN.”			Child care center	I, T, PS	East
“We have a form that is distributed throughout the year…concerning the need for food.”			Child care center	T, PS, SA	Shelby
“Done in more of a discrete way per applications…about what types of foods the child likes/dislikes.”			Child care center	I, T, PS, SA	Upper Cumberland
“On their application….there is an income question.”			Child care center	T, PS	South Central
“A parent needs assessment each fall…and ask the parent to indicate if they are vulnerable…”			Child care center	I, T, PS	Southeast
“Questionnaire”			Child care center	I, T, PS	Shelby
“CACFP form”			Child care center	I, T, PS	Mid Cumberland
“Forms”			Child care center	I, T, PS	Mid Cumberland
“We have a resource board available to parents…it has financial resources, food banks….”			Child care center	I, T, PS, SA	Southwest
Observational screenings	317	52.8 (54.8)			
“We make sure they do not act overly hungry during their time with us.”			Child care center	I, T, PS	East
“I have observed children that were undernourished….I have observed children.…who acted as if they had not eaten in a while.”			Group child care home	I, T, PS, SA	South Central
“I screen their lunch boxes daily to make sure there’s adequate food for the day.”			Group child care home	PS	East
“When we know we send home food….”			Child care center	I, T, PS, SA	Shelby
“Family does have food stamps but does run out of food.”			Other	PS	Southwest
“Families First is often a good indication of poverty….several families {are} enrolled at our center…”			Child care center	I, T, PS, SA	South Central

CACFP is the child and adult care food program

*I* infant, *T* toddler, *PS* pre-school, *SA* school-age

To explore differences in child care programs who received CACFP funds and programs who conducted food insecurity screenings, a chi-square analysis was performed with only categorical responses (e.g. yes/no for both variables). Assumptions were tested and Fisher’s exact test was used as observed counts of less than five were found in one cell. The chi-square results indicate that child care programs who receive CACFP funds differ significantly on whether or not they screen households for food insecurity $\chi$^2^ (1, n = 237) = 16.93, *p* ≤ 0.001 (Table 4). Ultimately, child care programs receiving CACFP funds are more likely to screen child care households for food insecurity.

**Table 4:** Chi-square analysis of child care programs receiving CACFP funds and screening of child care households for food insecurity (n = 237)

Variable	n	Child care programs who receive CACFP funds		$\chi$^2^	*p*
		Yes	No		
Child care programs who screen households for food insecurity				16.93	< 0.001**
Yes	25	23 (18.25%)	2 (1.80%)		
No	212	103 (81.75%)	109 (98.20%)		
Totals	237	126 (53.46%)	111 (46.84%)		

CACFP is the child and adult care food program

**Statistically significant at *p* < 0.05

From the initial survey, 122 child care programs provided their email addresses for the follow-up survey. Forty-one programs completed the follow-up survey, a 33.61% response rate. When asked if child care programs would consider including two questions assessing the risk of food insecurity on household admissions paperwork, 63.42% (n = 26) indicated they would consider, 7.31% (n = 3) would not consider, and 29.27% (n = 12) were neutral.

Child care programs also provided open-ended responses on follow-up survey questions about general food insecurity information the researchers should know, any burdens food insecurity places on child care families, and possible resources needed to support child care families experiencing food insecurity. Thematic elements from the open-ended questions and frequency counts (n = 41) are presented in Table 5. Some child care program directors described little to no food insecurity experienced by their families (e.g., “This is not a problem or even a struggle with the families at my center.”) and subsequently no associated burdens (e.g., “This does not really impact our families.”). Conversely, other directors detailed the worry, stress and burden food insecurity placed on families including having to choose between food and essential needs such as gasoline, medicine, and clothing, or even which types of foods to buy (e.g., “healthy” versus “less healthy”). Needs identified by child care directors for food insecure families were seemingly two-fold and included educational resources about purchasing healthy foods while on a budget and a list of available community resources (e.g., food banks, pantries, weekend food bags, etc.). Importantly, directors mentioned the reluctance that families feel in asking for help with food access noting it is a “private” issue.

**Table 5:** Emerging themes of child care programs’ perspectives on food insecurity (n = 41)

Thematic elements	Sample quotes from child care program directors	n	%
Question: What should the researchers know about food insecurity for your child care households?			
Theme: Food insecurity is not a problem or issue for our families	“Most of the children in our facility do not face this issue in their home.” “I doubt that our families have food insecurities.” “We currently do not have any insecurities.” “The families we serve are all in a position to afford enough food for their children.” “I am not aware of any families in need of food at this time.” “This is not a problem or even a struggle with the families at my center.” “This does not really impact our families.” “I have not seen a real issue with food insecurity.”	12	29.27
Theme: Families need extra help from the facility and/or other sources	“There have been occasions where we give families bags of groceries from our church pantry……” “We have families that *{do}* not qualify for food stamps but do need extra help to feed their family.” “Some of our families are seeking pantries and other ways to get food because of the lack of funds for food.” “Some parents gets food stamps therefore we all try to give well balanced meals.” “We do have families in our center who do not know where the next meal is coming from. We do what we can by offering extras to go home with them.”	8	19.51
Theme: There is a lack of high quality foods or healthy options	“The children do not always get proper nutrition.” “Healthy food is expensive and a lot of our families may opt for a can of stew or frozen pizza. Others eat a lot of pasta or potatoes.” The families we serve are all in a position to afford enough food for their children. This does not mean they are making consistently healthy choices for their children “The availability and cost of nutritious foods is a concern for low-income families. Fresh, organic, nutritious foods are expensive. Families often choose lesser quality of foods to stretch their dollars.”	6	14.63
Theme: Families make choices impacting nutrition	“….this does not mean they are making consistently healthy choices for their children.” “Some families feel they cannot afford fresh non-processed food; therefore they purchase less healthy food choices.” “….families in my child care program have access to adequate nutritious food but lead such a busy life that they rely on fast food or snacks.” “The availability and cost of nutritious foods is a concern for low-income families. Fresh, organic, nutritious foods are expensive. Families often choose lesser quality of foods to stretch their dollars.”	5	12.20
Theme: Families are reluctant to ask for help	“They are proud and do not want to admit that they need help.” “Families are reluctant to share their needs.” “It is a very private, yet real issue and many are not comfortable even displaying it….”	3	7.31
Question: What are the burdens of food insecurity for child care families?			
Theme: Financial issues force families to choose	“It is a choice between having gas money to go to work or having enough food in the home to feed the family.” “….food choices (pop-tarts, drink mixes, etc.), no fruits or vegetables normally” “Cost of purchasing healthy foods, for example fruit punch at $1.99 per 1/2 gallon and $3.99 for 100% fruit juice.” “It leads to making decisions to let one need go; decisions have to be made to let important needs go.” “The choice between food and other essentials such as clothing, medicines, etc.”	9	21.91
Theme: Food insecurity is not a burden for our families	“At this time, I am unaware of any needs.” “I don't really have any families that I know of that this applies to.” “This does not really impact our families.” “This is not a problem the families seem to have.”	8	19.51
Theme: Food insecurity adds worry or stress to our families	“Worry and uncertainty.” “Stress about having enough money to budget for healthy food.” “Worry about meeting the needs of their child, trying to choose what to spend their money on.” “It would have added stress to the family and the children.” “Parents are worried, upset, or stressed.”	8	19.51
Theme: Families have limited knowledge of proper nutrition	“…lack of skills in reading labels and ability to do comparative shopping.” “Parents have hard time meeting requirements for food groups.” “The purchase of non-nutritious food is a problem, without proper education on how to purchase the right food is a problem.” “The families are not eating healthy due to lifestyle.” “Families often choose fast food options verses having nutritious foods available at homes.”	8	19.51
Question: What resources are needed for food insecure child care families?			
Theme: Address immediate food needs	“I would love to have grocery vouchers/gift cards available ….” “Food shelf (no cost) located at the center or beside…” “We direct them to food banks.” “I’d love to offer weekend food bags as well as food I can serve to them daily.” “Hermetically sealed food items for snacks for those children that may request additional food; refer to local food pantries.” “I would like to have a program like the backpack food program where the children are able to take food home over the weekend.” “We have a food basket at the front of our center offering free food to families in need.” “Access to more food banks.” “Daily food bag to take home.”	14	34.15
Theme: Educational programs or training are needed	“… there was a program ….on making healthy, low-cost meals. They also taught families how to make meals with what was in the cupboard.” “Education on how to plan meals, prepare and purchase fresh foods on a budget.” “Educational programs on how to purchase the right kind of food would be helpful.” “Parent training in how to purchase, how to prepare good meals and training in benefits of making good food choices.”	7	17.01
Theme: A list of resources are needed	“….refer to local food pantries.” “….. a list of resources would be helpful.” “Community resources and direct contact numbers or emails to give families who this may be an issue they are having.” “Would like a list of available resources to help families in need.”	6	14.63
Theme: No resources are needed at this time	“None at this time.” “None.” “No resources needed at this time.”	6	14.63

## Discussion

The use of a representative sample of licensed Tennessee child care programs (N = 272), both geographically and program type, suggests the ability to generalize study findings to programs across Tennessee and potentially other states. This study included the collecting and reporting of primary data, which is essential in moving towards an increased understanding of food system equity (Rodman-Alvarez & Colasanti, 2019).

A key research finding, meeting the first study objective, discovered that less than 10% of sampled child care programs are screening for food insecurity. Programs who screened, described their screening practices in three main methods: verbal, written and observational (Table 3). Informal screening methods, verbal and observational, included the widest variety of screening efforts (e.g. face-to-face conversations with families, observations of children’s hunger, etc.). Written screenings, a more formalized screening method, were based on information from forms or questionnaires and were the most frequently reported method used by child care directors. Notably, written screenings are based on information reported directly by households, while observational and to some extent verbal screenings are evaluated by a child care director’s observation or through the interpretation of a verbal conversation.

When exploring differences between programs who received CACFP funding and those who screened for food insecurity, the second study objective, significant differences were found. Programs receiving CACFP funds were significantly more likely to screen for food insecurity. Just over half (56.99%) of child care programs in this study reported they received CACPF funds, which is similar to a 2015 study where 49.85% of child care programs received CACFP funds (Heflin et al., 2015).

Nearly 75% of sampled programs were identified as child care centers and 22% were home-based centers, yet less than 60% of overall programs received CACFP reimbursement funds. The child care centers could be eligible for CACFP funds but would need to “determine the reimbursement for each child from that child’s household income” (Gordon et al., 2011). The home-based centers could receive tiered CACFP reimbursements “determined by a mix of factors including neighborhood, provider and family income” (Heflin et al., 2015). Importantly, programs participating in the CACFP “may reduce the burden on the household food supply because their parents do not need to send food from home for them to eat while they are in care,” suggesting an improvement in food access and consequently a reduction in household food insecurity (Heflin et al., 2015).

Lastly, child care programs recognize the difficulties that food insecurity can place on households (Table 5). These burdens have been described as “difficult tradeoffs” between buying food and other items such as medications or utilities (Frank et al., 2006; Holben & Marshall, 2017; Nord & Kantor, 2006; Sullivan et al., 2010). There may be a disconnect between how a child care director perceives food insecurity of their enrolled families versus the reality for those families as evidenced by programs who indicated there were no issues with food insecurity, yet received CACFP funds. One explanation may be that “low-income children receiving care in centers are estimated to be less likely to participate in the CACFP if they live in higher income areas” suggesting that a center in a higher income area should explore participating in the CACFP if they suspect they could meet the CACFP income thresholds needed for their particular type of care (Gordon et al., 2011; Heflin et al., 2015). Determining the level of food insecurity in a child care setting can be difficult because programs in low-income geographic areas accept children from higher income areas and vice versa. This geographic variation underscores the importance of child care facilities having screening procedures in place to assess the risk for household food insecurity and identify families who may help qualify a program for the CACFP reimbursement funds.

One limitation of the study was the collection of self-reported data which is difficult to validate. Another limitation was that survey inclusion criteria required the role of “program director” or “assistant director” and if participants considered themselves the child care “owner” then they were excluded from the sample.

## Conclusions

The current study focused on documenting the prevalence of Tennessee child care programs screening for food insecurity and found that only about 9% (n = 25) of programs were. Moving forward, child care programs should be viewed as a partner in food insecurity screening efforts. Programs indicate a willingness to do so, with approximately 10% (n = 26) of child care directors reporting that they would consider adding food insecurity screening questions to admissions paperwork.

Another emphasis of the study was to investigate differences in child care programs who screen for food insecurity and receive CACFP reimbursement funds. Programs receiving CACFP funds were found to be significantly more likely to screen child care households for food insecurity and those screening methods (e.g. verbal, written, and observational) vary between programs.

The last study priority was to explore possible burdens food insecurity may place on families as reported by child care directors. Many directors do not perceive food insecurity is an issue for their child care families; however, they do recognize that households who struggle with food insecurity have significant financial, physical and emotional burdens. In the future child care programs can take a lead role to act “as a natural access point at which interventions can reach low-income children, perhaps those children whose parents would not sign up for nutritional assistance directed at families” (Gordon et al., 2011).

In summary, study results highlight the importance of examining the intersection of child care and food insecurity. Future research should focus on validating food insecurity screening questions within child care families themselves in order to better understand their household experiences and needs. Gaining a deeper understanding into the role that child care and nutrition programs, like the CACFP, play in alleviating food insecurity can better help child care directors or policy makers implement systems that best support families.
